# Knockdown of circular RNA tousled-like kinase 1 relieves ischemic stroke in middle cerebral artery occlusion mice and oxygen-glucose deprivation and reoxygenation-induced N2a cell damage

**DOI:** 10.1080/21655979.2021.2024684

**Published:** 2022-01-22

**Authors:** Rile Wu, Qiang Yun, Jianping Zhang, Zhong Wang, Xiaojun Zhang, Jingang Bao

**Affiliations:** Department of Neurosurgery, Inner Mongolia People’s Hospital, Hohhot, China

**Keywords:** CircTLK1, miR-26a-5p, PTEN, IGF-1R, GLUT1, ischemic stroke, middle cerebral artery occlusion, oxygen-glucose deprivation and reoxygenation

## Abstract

Ischemic stroke (IS) is an essential contributor to the neurological morbidity and mortality throughout the world. The significance of circular RNA tousled-like kinase 1 (circTLK1) in IS has been documented. This study set out to explore the mechanism of circTLK1 in IS. Middle cerebral artery occlusion (MCAO) mouse models *in vivo* and oxygen-glucose deprivation and reoxygenation (OGD/R) cell models *in vitro* were first established, followed by evaluation of infarct volume and neurological impairment, and cell viability and apoptosis. The expression patterns of circTLK1, miR-26a-5p, phosphatase and tensin homolog (PTEN), insulin-like growth factor type 1 receptor (IGF-1 R), and glucose transporter type 1 (GLUT1) were detected by RT-qPCR and Western blotting. Co-localization of circTLK1 and miR-26a-5p in N2a cells was tested by fluorescence in situ hybridization assay. The binding relationships among circTLK1, PTEN, and miR-26a-5p were verified by dual-luciferase assay and RNA pull-down. circTLK1 and PTEN were highly expressed while miR-26a-5p was under-expressed in IS models. circTLK1 knockdown decreased infarct volume and neurological impairment in MCAO mouse models and relieved OGD/R-induced neuronal injury *in vitro*. circTLK1 and miR-26a-5p were co-located in the N2a cell cytoplasm. circTLK1 regulated PTEN as a sponge of miR-26a-5p. PTEN positively regulated IGF-1 R and GLUT1 expressions. miR-26a-5p inhibitor annulled the repressive effects of circTLK1 silencing on OGD/R-induced neuronal injury. sh-PTEN partially annulled the effects of the miR-26a-5p inhibitor on OGD/R-induced neuronal injury. In conclusion, circTLK1 knockdown relieved IS via the miR-26a-5p/PTEN/IGF-1 R/GLUT1 axis. These results may provide a new direction to IS potential therapeutic targets.

## Graphical abstract

**Figure:**
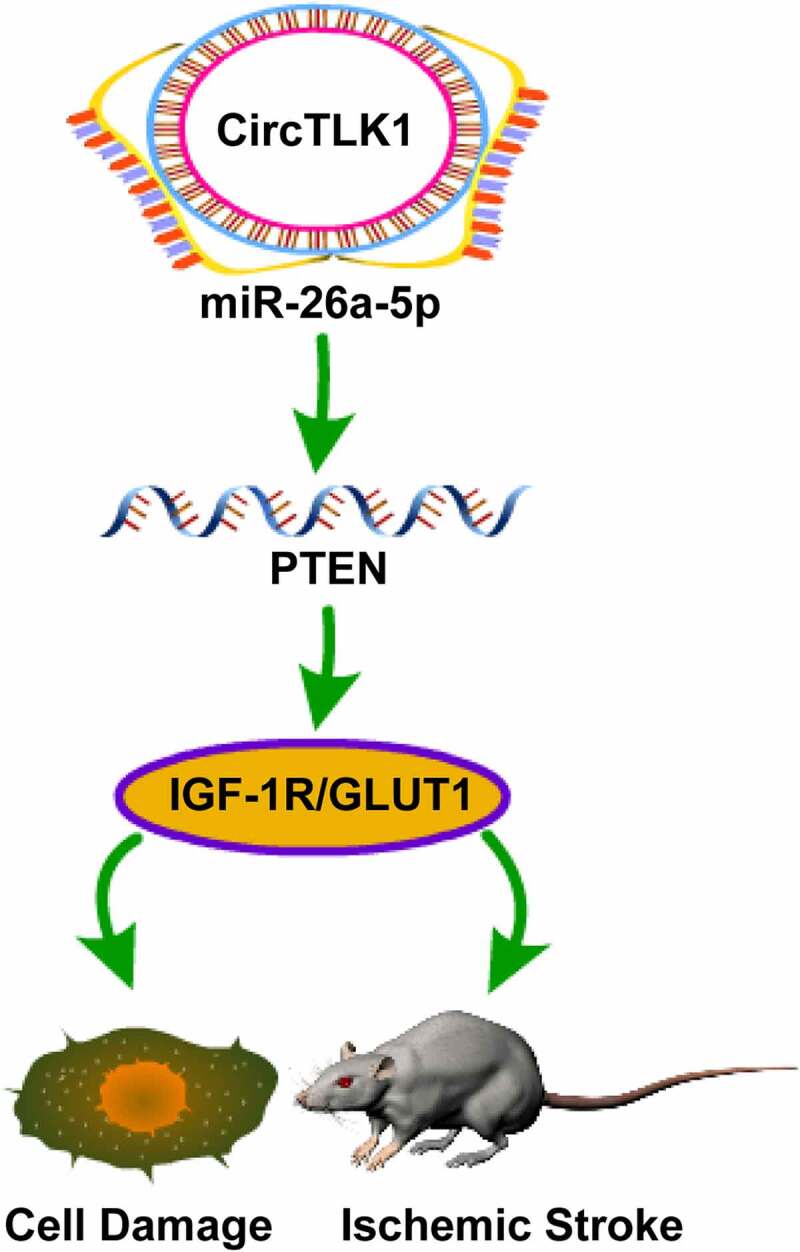

## 1. Introduction

Stroke is a type of clinical syndrome caused by the systemic or local damage of the brain that lasts for more than 24 h or leads to deaths, which is the most serious cardiovascular syndrome and even more dreaded than myocardial infarction and heart failure, resulting in approximately 9% of the death of the global population [1–3]. Stroke can be classified as hemorrhagic, due to bleeding, or ischemic, due to the lack of blood flow [4] Ischemic stroke (IS) is responsible for a majority of strokes, accounting for more than 80% of all strokes [5,6]. IS is mainly attributed to an embolus or thrombus blocking part of the brain blood supply, which leads to the death of brain nerve cells [7]. To prevent strokes, the management of main risk factors is necessitated, including hyperlipidemia, diabetes mellitus, hypertension, antithrombotic therapy, and tobacco exposure [8]. Despite the improvement in the early diagnosis and effective treatments of vascular risk factors, stroke is still a major contributor to long-term disability and mortality all over the world and there is still no effective treatment for neuronal damage caused by ischemia [9,10]. Owing to the complex pathogenesis of IS, it is of vital importance to study its pathophysiological mechanism and explore new potential therapeutic targets.

Circular RNAs (circRNAs), featured by the back-splicing due to the covalently closed continuous loops, are the endogenous non-coding RNAs, some of which are highly expressed in the brain and play roles in a variety of diseases in the central nervous system [11]. Accumulating evidence suggests that the dysregulation of circRNA expression pattern is involved in IS pathological process [11–14]. circRNA tousled-like kinase 1 (circTLK1) derived from TLK1 mRNA backsplicing, is located on chromosome 2: 171,884,848–171,902,872 and is at a length of 256 nucleotides [15]. The involvement of circTLK1 in IS has been documented once so far [16]. However, the specific molecular mechanism of circTLK1 in regulating IS remains elusive. microRNAs (miRNAs) are the non-coding RNAs including 18 to 22 nt, which have interactions with circRNAs and also show regulation on the expression levels of downstream messenger RNAs (mRNAs) [17,18]. miRNAs are important regulators of the pathological process of IS [18,19]. The involvement of miR-26 has been shown in the pathophysiology of cerebrovascular diseases [20]. The Starbase bioinformatics software predicted that phosphatase and tensin homolog (PTEN) might be a downstream target of miR-26a-5p. It has been reported that PTEN can modulate the neuron apoptosis and neurites outgrowth via the PI3K/Akt/mTOR pathway [21]. Additionally, PTEN regulates insulin-like growth factor type 1 receptor (IGF-1 R)-mediated drug resistance in melanoma [22]. PTEN can dephosphorylate AKT and downregulate the expression of glucose transporter type 1 (GLUT1) on the serosa of cancer cells [23]. IGF-1 R regulates the expression of GULT by phosphorylating AKT [24]. However, at present, there is no report on whether PTEN can affect IS progression by mediating IGF-1 R/GLUT1.

Based on previous studies, we proposed the hypothesis that circTLK1 affected the progression of stroke by regulating the miR-26a-5p/PTEN/IGF-1 R/GLUT1 axis. This study sought to explore the effects of circTLK1 on IS and its related molecular mechanism, hoping to provide a novel target option for IS diagnosis and treatment.

## 2. Materials and methods

### 2.1. Ethics statement

All procedures were authorized by the academic ethics committee of Inner Mongolia People’s hospital and strictly followed the 8^th^ version of Guide for the Care and Use of Laboratory Animals in 2011. All efforts were made to reduce the pain of mice.

### 2.2. Experimental animals

Healthy male C57BL/6 J mice (7–8 weeks) provided by Beijing Vital River Laboratory Animal Technology (Beijing, China) were adaptively fed for 1 week before the experiment at 24–26°C and 50–60% humidity with ad libitum access to water and food.

### 2.3. Middle cerebral artery occlusion (MCAO) model establishment

The mice were randomly assigned to the sham group, MCAO group, MCAO + sh-NC group, and MCAO + sh-circTLK1 group (N = 18/group). The MCAO model was established following the reported methods after appropriate modifications [25]. The mice fasted but were fed with available water for 12 h before operation. All fasted mice were intraperitoneally injected with 2% pentobarbital (50 mg/kg) (B5646, APExBIO, Houston, TX, USA). After that, the middle of the neck was cut open and the left common carotid artery (CCA), external carotid artery (ECA), and internal carotid artery (ICA) were separated. Subsequently, the CCA and ECA were ligated and a small area was slowly cut at the bifurcation of CCA. Next, the MCAO threaded plug (1623, Cinontech, Beijing, China) was inserted into the ICA at 18–20 mm until feeling no resistance. After 2 h of the occlusion, the threaded plug was pulled out and the wound was sutured. After 1 h of MCAO, reperfusion was performed for 24 h. After neurobehavioral scoring in all mice, mice were euthanized and mouse brain tissues were removed for further experimentation, with 6 mice used for 2,3,5-Triphenyltetrazolium chloride (TTC) staining, 6 for mice used terminal deoxynucleotidyl transferase dUTP nick end labeling (TUNEL) staining and immunohistochemistry, and 6 mice used for reverse transcription quantitative polymerase chain reaction (RT-qPCR) and Western blot (WB) after tissue homogenization.

During the operation, a thermostatic blanket was used to maintain mouse rectal temperature at 37°C. All operations except MCAO were performed on the mice in the sham group. The sh-NC and sh-circTLK1 (GenePharma, Shanghai, China) were injected into the lateral ventricle 1 day prior to MCAO.

### 2.4. Neurological impairment assessment

After 1 day of MCAO modeling in mice, the neurological injury of the mice was evaluated using the 6-point scale, and the cerebral infarction was assessed using the TTC staining [26]. The brain neurological impairment scores were judged based on the following criteria: 0, no significant deficits; 1, failure to entirely stretch the left forepaw when the tail of rats was stimulated; 2, contralateral circling to the left when the tail of rats was stimulated; 3, walking or circling to the left; 4, walking only when they were stimulated; 5, unresponsiveness to stimulation with low-level consciousness.

### 2.5. TTC staining

Following neurological impairment assessment, the mice were euthanized using 50 mg/kg pentobarbital sodium, and the brains were rapidly collected, cut into sections at 2 mm, and cultured with 2% TTC solution (Sigma-Aldrich, St. Louis, MO, USA) at 37°C for 10 min. Next, the brain tissues were fixed with 4% paraformaldehyde for 1–2 days and scanned using a scanner, and the infarct volume was calculated using the Image J software (Media Cybernetics, Rockville, MD, USA). Brain tissue showed red when TTC reacted with dehydrogenase in normal tissues, while in ischemic brain tissues showed white because the dehydrogenase activity was reduced.

### 2.6. TUNEL staining

Cell apoptosis was examined using the in situ cell death detection kits (Roche, Indianapolis, IN, USA) [18]. After treatment, the cells were cultured with 0.2% Triton X-100 for 5 min and incubated with TUNEL reaction mixture containing TdT. Subsequently, the cells were cultured with 0.3% H_2_O_2_ for 10 min and stained with 0.5 μg/mL 4ʹ, 6-diamino-2-phenylindole (DAPI) for 5 min at room temperature. In TUNEL staining, green fluorescence represented apoptotic cells, and in DAPI staining, blue fluorescence represented the nucleus. The cells were observed using a fluorescence microscope (Olympus, Tokyo, Japan) and 5 visual fields were arbitrarily selected from each specimen.

### 2.7. Immunohistochemistry

The immunohistochemistry procedures were carried out according to the previous method with appropriate modifications [27]. The tissue sections were incubated with PTEN antibody (ab170941, 1:100) and stained with horseradish peroxidase (HRP) anti-rabbit immunoglobulin G and diaminobenzidine. Next, the sections were incubated with secondary antibody and stained with EnVision G2 System/AP Rabbit Mouse (Permanent Red) (Dako, Glostrup, Denmark). Lastly, the sections were counterstained with hematoxylin and observed under a fluorescence inverted microscope (Hitachi Limited, Tokyo, Japan).

### 2.8. Cell culture and oxygen-glucose deprivation reoxygenation (OGD/R) model

Mouse Neuro-2a (N2a) neuroblastoma cells provided by Cell Bank of Chinese Academy of Sciences (Shanghai, China) were cultured in the Dulbecco’s modified Eagle medium in combination with 10% fetal bovine serum (FBS) (Invitrogen, Carlsbad, CA, USA), 2 mM glutamine, 100 μg/mL streptomycin (Invitrogen), and 100 U/mL penicillin (Invitrogen) at 37°C with 5% CO_2_. In regard to OGD treatment, the cells were cultured in glucose-free deoxygenated Hanks’ balanced salt solution (Invitrogen) under hypoxic conditions (5% CO_2_, 95% N_2_) at 37°C for 3 h, then transferred to the normal culture medium, and cultured under normal oxygen (5% CO_2_) conditions at 37°C for 24 h [28].

### 2.9. Cell transfection

sh-circTLK1, sh-PTEN (negative control sh-NC), pcDNA3.1, pc-circTLK1, inhibitor NC, miR-26a-5p inhibitor, mimic NC, and miR-26a-5p mimic were designed and purchased from the GenePharma company. Cells were transfected using Lipofectamine 3000 (Invitrogen).

### 2.10. Cell counting kit-8 (CCK-8)

After transfection for 48 h, the cells were seeded into 96-well plates at a density of 2 × 10^4^ cell/well, incubated at 37°C containing 5% CO_2_ overnight, and supplemented with 10 μL CCK-8 solution in each well, followed by 4-h incubation at 37°C. Thereafter, the optical density value was read at 450 nm using an Elx800 Reader (Bio-Tek, Winooski, VT, USA) to assess the cell viability [18].

### 2.11. Flow cytometry

After transfection, the OGD/R-treated N2a cells were incubated for 48 h and cell apoptosis was assessed [18]. After incubation, the cells were stained with fluorescein isothiocyanate Annexin V apoptosis detection kits (BD Biosciences, Bedford, MA, USA) and cell apoptosis was determined using flow cytometry.

### 2.12. RT-qPCR

The total RNA content was extracted from tissues and cells using the Trizol reagent (Invitrogen) and reverse-transcribed into cDNA using the PrimeScript RT kits (Takala, Dalian, Liaoning, China). The qPCR was conducted using SYBR Premix Ex Taq II (Takala) on the 7500 Real-Time PCR system (ABI, Foster City, CA, USA). The expression patterns of circTLK1, miR-26-5p, PTEN, IGF-1 R, and GLUT1 were normalized to β-Actin and U6 levels. The results were calculated using the 2^−ΔΔCT^ method [29]. Primer sequences were circTLK1, 5’-CAGTCAATGGAGCAGAGAA-3’ (forward) and 5’-CCATTCTTGTTGCCTTTTTG-3’ (reverse); miR-26a-5p, 5’-TGCGCAACATCACTGCAAGTCT-3’ (forward) and 5’-CCAGTGCAGGGTCCGAGGTATT-3’ (reverse); PTEN, 5’-GTGCAGATAATGACAAG-3’ (forward) and 5’-GATTTGACGGCTCCTCT-3’ (reverse); IGF-1 R, 5’-AAACGCTGACCTCTGTTACCTCTC-3’ (forward) and 5’-GCGGATGAAGCCTGATGGAC-3’ (reverse); GLUT1, 5’-CAATCAAACATGGAACCACCG-3’ (forward) and 5’-CGATTGATGAGCAGGAAGCG-3’ (reverse); β-actin, 5’-CATCCGTAAAGACCTCTATGCCAAC-3’(forward) and 5’-ATGGAGCCACCGATCCACA-3’ (reverse); U6, 5’-CTCGCTTCGGCAGCAC-3’ (forward) and 5’-ACGCTTCACGAATTTGC-3’ (reverse).

### 2.13. WB

Following the previous WB procedures [29], the total protein was isolated from brain tissues and cells using the radioimmunoprecipitation assay lysis buffer containing protease inhibitor. The protein concentration was determined using bicinchoninic acid kits (BOSTER, Wuhan, Hubei, China). Next, the protein (30 μg) was isolated using 10% sodium dodecyl sulfate-polyacrylamide gel electrophoresis and then transferred to the polyvinylidene fluoride membranes (Millipore, Bedford, MA, USA). After that, the samples were blocked using 5% skim milk for 2 h and incubated overnight with following primary antibodies PTEN (ab170941, 1:1000), IGF-1 R (ab131476, 1:500), GLUT1 (ab14683, 1:2000), Bax (ab3191, 1:1000), Bcl-2 (ab196495, 1:1000), Cleaved caspase-3 (ab2302, 1:1500), and β-actin (ab8227, 1:1000) (all antibodies were acquired from Abcam, Cambridge, MA, USA) at 4°C. After washing with Tris-buffered saline-Tween 20 (TBST), the samples were incubated with HRP-labeled goat anti-rabbit secondary antibody IgG (ab205718, 1:2000) at room temperature for 1 h. The enhanced chemiluminescence working fluid (EMD Millipore, Billerica, MA, USA) was employed for development. With β-actin as the internal control, the gray levels of the band in WB images were quantified using the Image J software (Media Cybernetics).

### 2.14. Fluorescence in situ Hybridization (FISH)

FISH was performed as previously described [30]. The subcellular localization of circTLK1 and miR-26a-5p was testified using the FISH kits (BIS-P0001, Bersin Biotechnology, Guangzhou, Guangdong, China). Cell slides were treated with digoxin-labeled circTLK1 and miR-26a-5p probe hybridization solution. After hybridization at 42°C for 16 h, the slides were soaked in the 2 × normal saline sodium citrate buffer and 70% ethanol for 3 min and stained with DAPI for 10 min. Then the slides were imaged using a Zeiss LSM880NLO confocal microscope (Leica, Microsystems, Germany).

### 2.15. Dual-luciferase assay

The assumed miR-26a-5p binding sites in circTLK1 and PTEN were predicted using the online tool StarBase (https://starbase.sysu.edu.cn/). The circTLK1-3ʹ untranslated region (3ʹ-UTR) fragments with wild-type (circTLK1-WT) and mutant (circTLK1-MUT) binding sites of miR-26a-5p were inserted into the pmirGLO luciferase vectors (E1330, Promega, Madison, WI, USA). Likewise, PTEN-3ʹ-UTR fragments with wild-type (PTEN-WT) and mutant (PTEN-MUT) binding sites of miR-26a-5p were inserted into the pmirGLO luciferase vectors. The N2a cells were co-transfected with circTLK1-WT/circTLK1-MUT and PTEN-WT/PTEN-MUT and miR-26a-5p mimic or its NC using Lipofectamine 3000. After transfection for 48 h, the cells were collected for dual-luciferase assay (Promega) with the luciferase activity detected using a SpectraMax L fluorometer (Molecular Devices, San Francisco, CA, USA).

### 2.16. RNA pull-down assay

RNA pull-down assay was performed with reference to the previous study [31]. Briefly, N2a cells (1 × 10^7^) were collected and lysed. The biotinylated miR-26a-5p probe was synthesized by GenePharma (Shanghai, China) and incubated with streptavidin agarose beads (Thermo Scientific, Waltham, MA, USA). The cell lysate of the miR-26a-5p probe or oligo probe (control) was incubated overnight at 4°C, the RNA complex bound to the beads was eluted with the washing buffer, and the enrichment of circTLK1 pulled down by miR-26a-5p probe was detected by RT-qPCR.

### 2.17. Statistical analysis

The data were presented as mean ± standard deviation (SD). GraphPad Prism 8 software (GraphPad Software Inc San Diego, CA, USA) was employed for mapping and data analysis. An independent *t*-test was used for data comparisons between 2 groups and one-way analysis of variance (ANOVA) was applied for multi-group data comparisons. Tukey’s test was applied for the post hoc test. The correlation was analyzed using the spearman method. A value of *P* < 0.05 was regarded statistical significance.

## 3. Results

This study intended to explore the properties of circTLK1 on IS and its related molecular mechanism, so as to provide a new target for the diagnosis and treatment of IS. Therefore, the hypothesis of this study was that circTLK1 affected the IS progression by regulating the miR-26a-5p/PTEN/IGF-1 R/GLUT1 axis. By establishing MCAO mouse models *in vivo* and OGD/R cell models *in vitro*, the results showed that the expressions of circTLK1 and PTEN were promoted in IS, while the expression of miR-26a-5p was diminished. Knockdown of circTLK1 reduced infarct volume, nerve injury score and alleviated nerve cell injury. circTLK1 and miR-26a-5p co-located in the cytoplasm of N2a cells. circTLK1 regulated PTEN as a competing endogenous RNA (ceRNA) of miR-26-5p. In terms of mechanism, knockdown of circTLK1 alleviated IS by regulating the miR-26a-5p/PTEN/IGF-1 R/GLUT1 axis.

### 3.1. circTLK1/miR-26a-5p/PTEN were dysregulated in IS models

**Figure 1.**
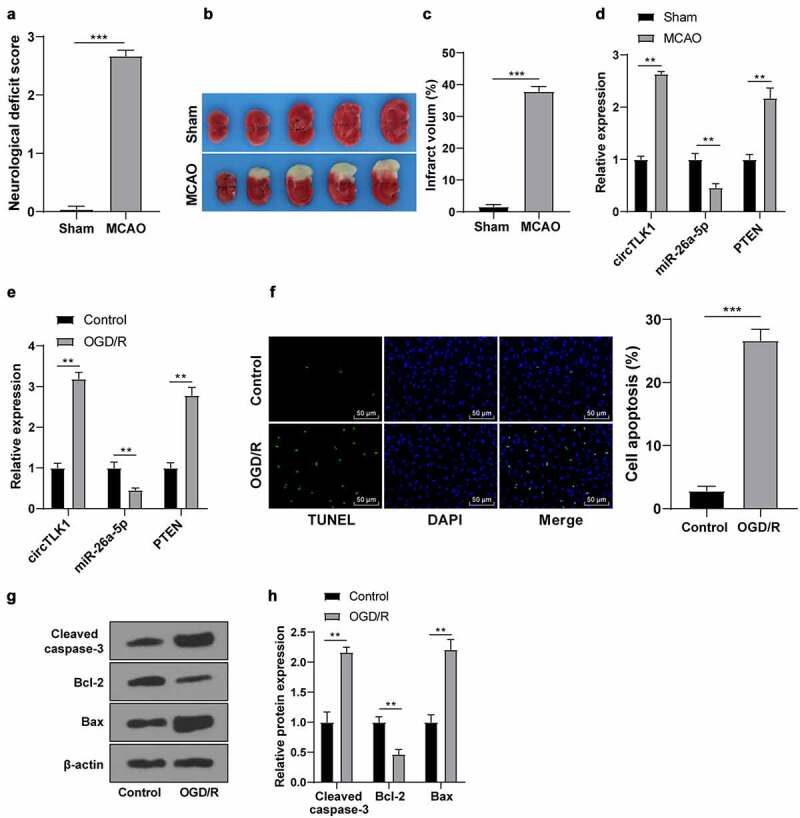
Expression of circTLK1/miR-26a-5p/PTEN in IS models *in vivo* and *in vitro*. MCAO (1 h/24 h) or sham operation was performed on C57BL/6 J mice; (a) Neurological impairment assessment, N = 18, *** *P* < 0.001; (b) TTC staining of brain sections; (c) Cerebral infarction volume.; (d) expressions of circTLK1, PTEN, and miR-26a-5p detected using RT-qPCR. N = 6, **P < 0.01, ***P < 0.001; (e-h) The N2a cells were treated by OGD/R treatment (3 h/24 h); (e) The expressions of circTLK1, miR-26a-5p, and PTEN were detected using RT-qPCR; (f) Apoptosis detected using TUNEL staining; (g-h) Cleaved caspase-3, Bcl-2, and Bax protein levels detected by WB. Cell experiment was conducted 3 times. Data were expressed as mean ± SD and analyzed by independent *t* test for data comparisons between 2 groups. **P < 0.01, ***P < 0.001.

A previous study has shown the dysregulated expression of circTLK1 in IS and its participation in IS progression [16]. To verify the circTLK1/miR-26a-5p/PTEN expression patterns in stroke, the MCAO models *in vivo* and OGD/R models *in vitro* were first established. In MCAO models, compared with the sham group, there were obvious neurological impairment (Figure 1a) and cerebral infarction (Figure 1b-c) in the MCAO group. Furthermore, upregulated circTLK1 and PTEN expression patterns and downregulated miR-26a-5p expression patterns were found in brain tissues of the MCAO group (Figure 1d). Subsequently, the N2a cells were induced by OGD/R for IS simulation *in vitro*. The trends of circTLK1, PTEN, and miR-26a-5p expression patterns were consistent with those *in vivo* (Figure 1e). Furthermore, TUNEL staining manifested increased OGD/R-treated cell apoptosis (Figure 1f). WB manifested that the protein levels of Cleaved caspase-3 and Bax in the OGD/R group were raised, while the protein level of Bcl-2 was decreased (Figure 1g-h). Briefly, circTLK1/miR-26a-5p/PTEN were dysregulated in IS models *in vitro* and *in vivo*, which might be involved in IS progression.

### 3.2. Silencing circTLK1 relieved neurological impairment induced by OGD/R

**Figure 2.**
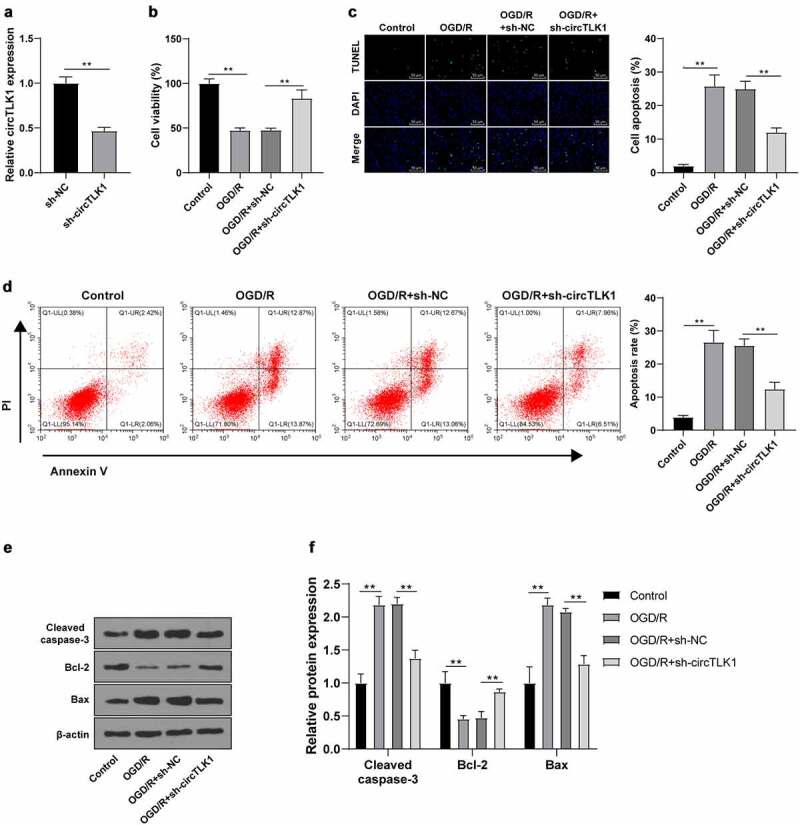
circTLK1 silencing relieved OGD/R-induced neurological impairment. (a) circTLK1 expression detected using RT-qPCR; (b) Cell viability detected using CCK-8; (c-d) Apoptosis detected using TUNEL staining and flow cytometry; (e-f) Cleaved caspase-3, Bcl-2, and Bax protein levels detected by WB. Cell experiment was conducted 3 times. Data were expressed as mean ± SD. Independent *t* test was used for data comparisons between 2 groups in panel A and one-way ANOVA was applied for data comparisons between multi-groups in panels B-F. Tukey’s test was used for post hoc test. **P < 0.01.

To study the effect of circTLK1 on cerebral ischemia, circTLK1 expression was knocked down using the shRNA technology to explore its function in OGD/R-induced N2a cell injury. RT-qPCR demonstrated that the circTLK1 in N2a cells was significantly downregulated after transfection of sh-circTLK1, indicating a successful transfection (Figure 2a). CCK-8 method showed significantly increased cell viability in OGD/R-induced N2a cells after circTLK1 silencing (Figure 2b). TUNEL staining and flow cytometry revealed significantly decreased OGD/R-induced apoptosis after circTLK1 silencing (Figure 2c-d). Furthermore, WB elicited that silencing circTLK1 partially averted OGD/R-increased protein levels of Cleaved caspase-3 and Bax and OGD/R-decreased protein levels of Bcl-2 (Figure 2e-f). Collectively, the knockdown of circTLK1 could relieve OGD/R-induced neurological impairment.

### 3.3. circTLK1 served as a miR-26a-5p molecular sponge

**Figure 3.**
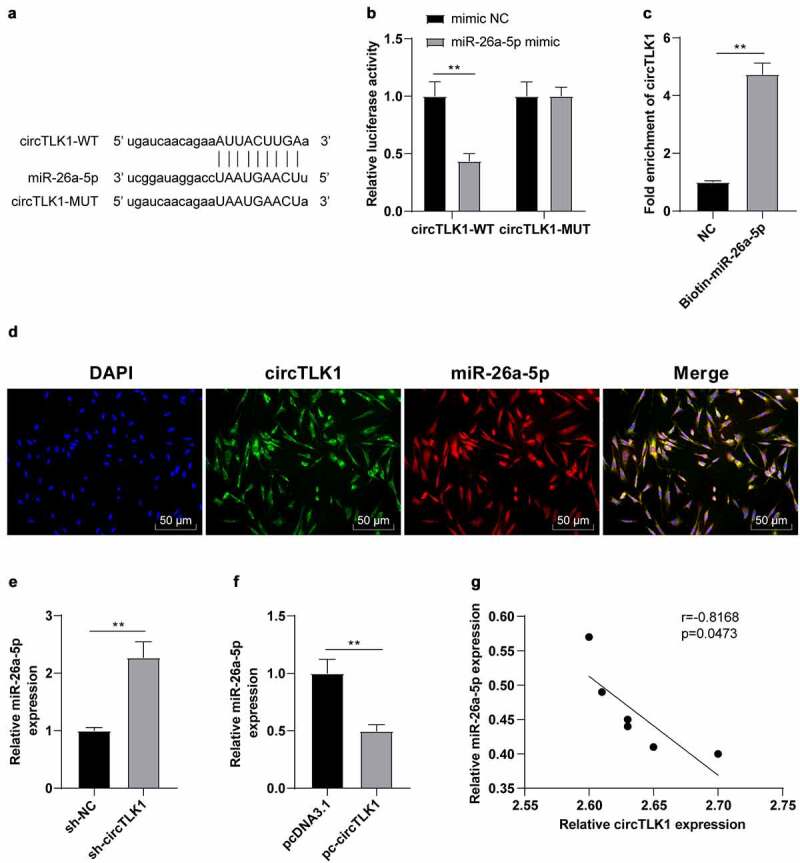
circTLK1 acted as a molecular sponge of miR-26a-5p. (a) potential binding sequence between circTLK1 and miR-26a-5p predicted using the StarBase; (b) binding relationship between circTLK1 and miR-26a-5p verified by dual-luciferase assay; (c) the interaction between circTLK1 and miR-26a-5p was evaluated by RNA pull down assay; (d) co-localization of circTLK1 and miR-26a-5p in N2a cells detected by FISH; (e-f) miR-26a-5p expression after sh-circTLK1 or pc-circTLK1 transfection detected using RT-qPCR; (g) correlation analysis between circTLK1 and miR-26a-5p expression in brain tissues of MCAO mice. Cell experiment was conducted 3 times. Data were expressed as mean ± SD and analyzed by independent *t* test. **P < 0.01.

The potential binding sites between circTLK1 and miR-26a-5p were predicted using the StarBase bioinformatics software (Figure 3a). After that, the dual-luciferase assay was employed to study whether circTLK1 could regulate miR-26a-5p. The relative luciferase activity was clearly repressed after co-transfection of miR-26a-5p mimic and circTLK1-WT, while the luciferase activity wasn’t apparently changed after the co-transfection of miR-26a-5p mimic and circTLK1-MUT (Figure 3b). Furthermore, RNA pull-down assay manifested that circTLK1 was enriched in biotin-labeled miR-26a-5p, which further proved the interaction between circTLK1 and miR-26a-5p (Figure 3c). FISH assay demonstrated that circTLK1 and miR-26a-5p were co-located in the cytoplasm of N2a cells (Figure 3d). miR-26a-5p is diminished in IS [28]. To explore whether miR-26a-5p was involved in IS by acting as the downstream miRNA of circTLK1, we conducted a series of experiments and manifested that compared with the cells transfected with sh-NC, the N2a cells transfected with sh-circTLK1 showed increased miR-26a-5p expression, while compared with the cells transfected with pcDNA3.1, the N2a cells transfected with pc-circTLK1 showed decreased miR-26a-5p expression (Figure 3e-f). Similarly, the results of correlation analysis manifested that there was a negative correlation between circTLK1 and miR-26a-5p expression in the brain of MCAO mice (Figure 3g). Overall, circTLK1 acted as a miR-26a-5p molecular sponge and negatively regulated miR-26a-5p expression.

### 3.4. miR-26a-5p targeted PTEN

**Figure 4.**
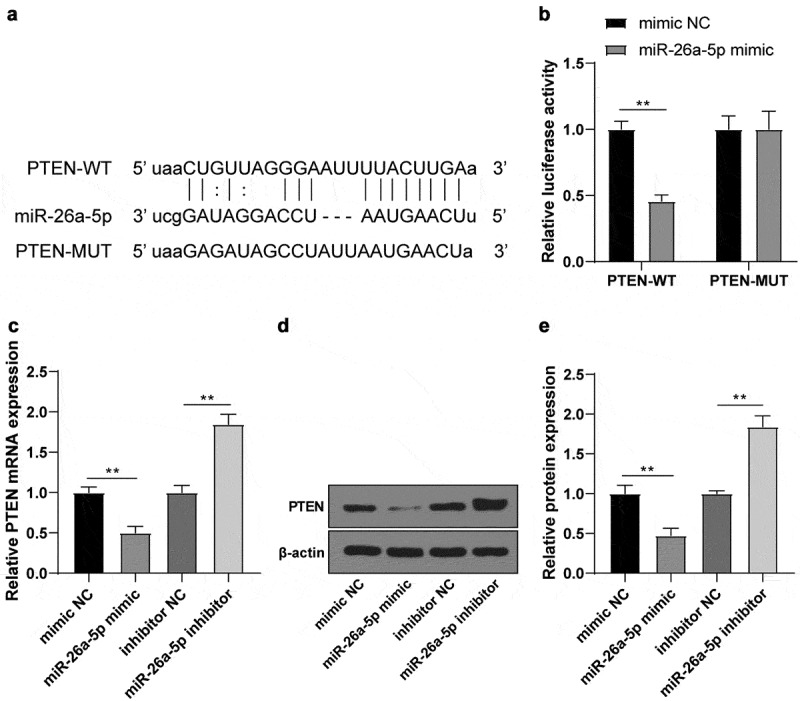
miR-26a-5p targeted PTEN. (a) potential binding sequence between miR-26a-5p and PTEN predicted using the StarBase; (b) binding relationship between miR-26a-5p and PTEN verified by dual-luciferase assay; (c) mRNA level of PTEN in N2a cells after transfection of mimic NC/miR-26a-5p mimic or inhibitor NC/miR-26a-5p inhibitor detected using RT-qPCR; (d-e) PTEN protein level in N2a cells after transfection of mimic NC/miR-26a-5p mimic or inhibitor NC/miR-26a-5p inhibitor detected by WB. Cell experiment was conducted 3 times. Data were expressed as mean ± SD and analyzed by independent *t* test. **P < 0.01.

PTEN is dysregulated in IS [32]. To further confirm whether PTEN affected IS progression by acting as the downstream target gene of miR-26a-5p, the potential binding sites between miR-26a-5p and PTEN were predicted using the StarBase bioinformatics software (Figure 4a). The binding relationship in N2a cells was identified by the dual-luciferase assay. The relative luciferase activity was significantly suppressed after co-transfection of miR-26a-5p mimic and PTEN-WT (Figure 4b). In addition, the PTEN levels were clearly reduced after transfection of miR-26a-5p mimic in N2a cells (Figure 4c-e), while the trends were reversed after transfection of miR-26a-5p inhibitor (Figure 4c-e). Thus, we concluded that miR-26a-5p targeted PTEN.

### 3.5 PTEN regulated IGF-1 R/GLUT1 expressions

**Figure 5.**
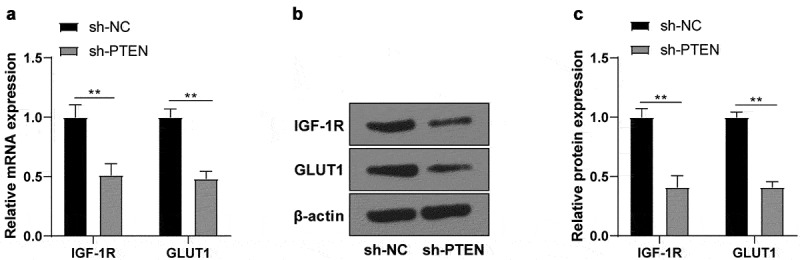
PTEN raised IGF-1 R/GLUT1 expression. (a) IGF-1 R/GLUT1 mRNA level in OGD/R-induced N2a cells after sh-PTEN transfection detected using RT-qPCR; (b-c) The protein level of IGF-1 R/GLUT1 in OGD/R-induced N2a cells after transfection of sh-PTEN detected by WB. Cell experiment was conducted 3 times. Data were expressed as mean ± SD and analyzed by independent *t* test. **P < 0.01.

PTEN can regulate IGF-1 R/GLUT1 in cancers [22,23]. To further confirm whether PTEN had the same effects on IS, the changes of IGF-1 R/GLUT1 mRNA and protein levels in OGD/R-induced N2a cells after transfection of sh-PTEN were detected using RT-qPCR and WB, which revealed that IGF-1 R/GLUT1 mRNA (Figure 5a) and protein (Figure 5b-c) levels were significantly decreased after PTEN knockdown.

### 3.6. circTLK1 regulated OGD/R-induced neurological impairment through the miR-26a-5p/PTEN/IGF-1 R/GLUT1 axis

**Figure 6.**
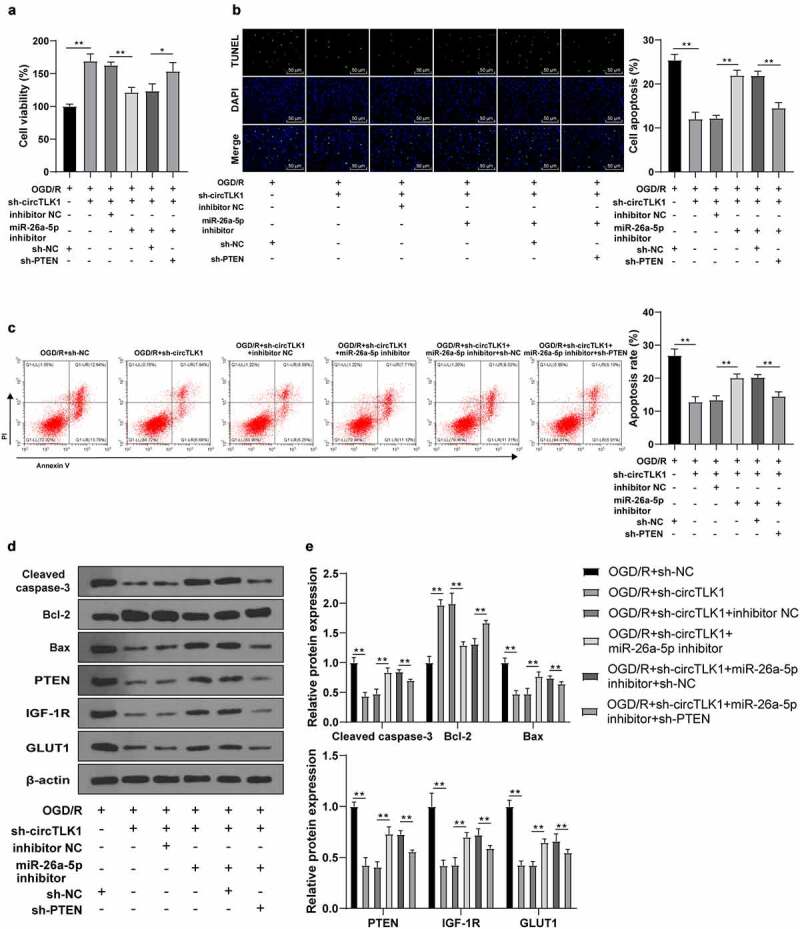
circTLK1 regulated OGD/R-induced neurological impairment via the miR-26a-5p/PTEN/IGF-1 R/GLUT1 axis. (a) N2a cell viability treated by OGD/R after transfection detected using CCK-8; (b) N2a cell apoptosis rate treated by OGD/R after transfection detected using TUNEL staining; (c) Apoptosis rate detected using flow cytometry; (d-e) Cleaved caspase-3, Bcl-2, Bax, PTEN, IGF-1 R, and GLUT1 protein levels detected by WB. Cell experiment was conducted 3 times. Data were expressed as mean ± SD. One-way ANOVA was used for data comparisons between multi-groups. Tukey’s test was employed for post hoc test. * *P* < 0.05, **P < 0.01.

To validate the potential effect of the circTLK1/miR-26a-5p/PTEN/IGF-1 R/GLUT1 axis on IS progression, the cell apoptosis and viability were detected. In OGD/R-treated N2a cells, silencing circTLK1 significantly promoted cell viability, while co-transfection of miR-26a-5p inhibitor inhibited cell viability; further transfection of sh-PTEN inhibited the repressive effects of miR-26a-5p inhibitor on cell viability (Figure 6a). TUNEL staining and flow cytometry revealed that the knockdown of circTLK1 significantly reduced cell apoptosis, while co-transfection of miR-26a-5p inhibitor stimulated cell apoptosis; further transfection of sh-PTEN partially reversed the effects of miR-26a-5p inhibitor on promoting cell apoptosis (Figure 6b-c). Consistently, WB manifested that the knockdown of circTLK1 significantly reduced Cleaved caspase-3 and Bax levels and promoted Bcl-2 levels, while these trends were reversed by miR-26a-5p inhibitor transfection; further transfection of sh-PTEN partially averted the effects of miR-26a-5p inhibitor. In addition, the levels of PTEN, IGF-1 R, and GLUT1 were significantly reduced after circTLK1 knockdown, while miR-26a-5p inhibitor partially reversed these trends; further transfection of sh-PTEN partially reversed the effects of miR-26a-5p inhibitor (Figure 6d-e). Altogether, these results suggested that circTLK1 regulated OGD/R-induced neurological impairment via the miR-26a-5p/PTEN/IGF-1 R/GLUT1 axis.

### 3.7. Knockdown of circTLK1 relieved IS in MCAO mouse

**Figure 7.**
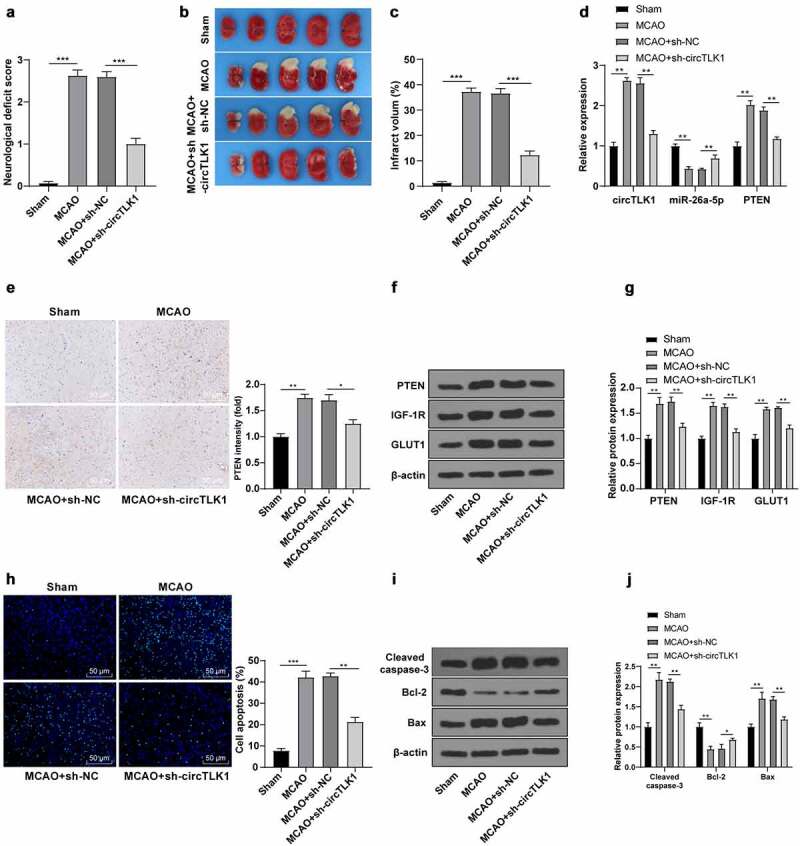
Knockdown of circTLK1 relieved IS in MCAO mice. (a-j) sh-NC or sh-circTLK1 was injected into mouse lateral ventricle. After 24 h of injection, mice were treated by MCAO (1 h/24 h) or sham operation and brain tissues were collected and analyzed; (a) Neurological impairment assessment, N = 18, *** *P*< 0.001; (b) TTC staining of brain sections; (c) Cerebral infarction volume; (d) circTLK1, miR-26a-5p, and PTEN expressions in brain tissues were detected using RT-qPCR; (e) PTEN expression in brain tissues detected using immunohistochemistry; (f-g) PTEN, IGF-1 R, and GLUT1 protein levels in brain tissues detected by WB; (h) Cerebral cortical apoptosis detected using TUNEL staining; (i-j) Cleaved caspase-3, Bcl-2 and Bax protein levels in brain tissues detected by WB. N = 6, *P < 0.05, **P < 0.01, ***P < 0.001.

Finally, the role of circTLK1 *in vivo* in the MCAO mouse models was assessed. Compared with the sham group, the rats in the MCAO group showed neurological impairment (Figure 7a) and cerebral infarction (Figure 7b-c), while these changes were clearly improved after knockdown of circTLK1 (Figure 7a-c). RT-qPCR and immunohistochemistry elicited that circTLK1 and PTEN were significantly elevated and miR-26a-5p was downregulated in mouse brain tissues of the MCAO group, while these trends were annulled after circTLK1 knockdown (Figure 7d-e). WB demonstrated that silencing circTLK1 suppressed the increase of PTEN, IGF-1 R, and GLUT1 levels in MCAO mice (Figure 7f-g). Furthermore, compared with the sham group, the apoptosis rate (Figure 7h) and Cleaved caspase-3 and Bax levels were increased and the Bcl-2 level (Figure 7i-j) was reduced in the MCAO group, while these trends were averted after knockdown of circTLK1 (Figure 7h-j). Conjointly, the effects of circTLK1 on IS were associated with the miR-26a-5p/PTEN/IGF-1 R/GLUT1 pathway.

## 4. Discussion

Stroke is a chief cause of disability and the 2nd greatest cause of mortality over the world, with an increasing incidence in developing countries [6]. Meanwhile, the hard-done work of our peers has shown that the IS is estimated to account for 87% of strokes [5]. circTLK1 was previously shown to be implicated in IS progression [16]. This study illustrated that circTLK1 silencing relieved IS in MCAO mice and OGD/R-induced N2a cells via the miR-26a-5p/PTEN/IGF-1 R/GLUT1 axis.

Various circRNAs are well-established to possess the ability to function in IS brain injury [33]. While there is also much evidence to highlight that miRNAs are key molecular mediators for IS and many other diseases [34,35]. A prior study has reported that PTEN can modulate the apoptosis of neurons [21]. Herein, we explored the circTLK1/miR-26a-5p/PTEN expression patterns in IS models. Our results uncovered elevated circTLK1 and PTEN expressions while repressed miR-26a-5p expression in brain tissues of MCAO mice and OGD/R-induced N2a cells. Consistently, a previous study showed the same trend that circTLK1 level was increased in brain tissues of focal cerebral ischemia-reperfusion mouse model [16]. The decreased miR-26a-5p level was detected in MCAO/reperfusion mouse and OGD/R-induced N2a cells [28]. PTEN expression was elevated in IS [36]. In conclusion, circTLK1/miR-26a-5p/PTEN was dysregulated in the *in* *vitro* and *in* *vivo* IS models.

To further study the effect of circTLK1 on IS, we knocked down the circTLK1 expression in OGD/R-induced N2a cells. The subsequent experimentation in our study revealed that OGD/R-induced N2a cell viability was enhanced, OGD/R-induced apoptosis was reduced, and the increased Cleaved caspase-3 and Bax protein levels and decreased Bcl-2 protein level induced by OGD/R were partially reversed upon the circTLK1 knockdown. The reduced protein level of Cleaved caspase-3 and the elevated protein level of Bcl-2 indicate a reduction in neuronal apoptosis [37]. In accordance, circTLK1 shRNA lentivirus treatment rescued the OGD/R-induced decrease in neuronal survival [16]. In summary, circTLK1 knockdown relieved OGD/R-induced cell injury.

There is evidence to demonstrate that lncRNA AK038897 aggravates ischemia/reperfusion (I/R)-induced cerebral injury by acting as a ceRNA for miR-26a-5p [28]. To study whether miR-26a-5p played a role in the progression of IS as a downstream miRNA of circTLK1, we predicted the potential binding sites between circTLK1 and miR-26a-5p using the StarBase bioinformatics software and verified the binding relationship by dual-luciferase assay. RNA pull-down assay validated the interaction between circTLK1 and miR-26a-5p. Our subsequent results showed that circTLK1 and miR-26a-5p were co-located in the N2a cell cytoplasm. sh-circTLK1-transfected N2a cells showed increased miR-26a-5p expression, while pc-circTLK1-transfected N2a cells showed decreased miR-26a-5p expression. To study the function of miR-26a-5p in IS, we knocked down circTLK1 and co-transfected miR-26a-5p inhibitor into OGD/R-treated cells. Our results unraveled that circTLK1 knockdown promoted viability, reduced apoptosis, and diminished PTEN, IGF-1 R, and GLUT1 expressions in OGD/R-treated cells, while miR-26a-5p inhibitor brought about reversed trends. Consistently, melatonin showed protective effects on OGD/R-induced PC12 cells by modulating miR-26a-5p expression to improve inflammation and autophagy and reduce cell apoptosis in I/R-induced cerebral injury, whereas the downregulation of miR-26a-5p neutralized melatonin’s protective effects [38]. In brief, circTLK1 acted as a miR-26a-5p molecular sponge and negatively regulated its expression.

PTEN has the function of modulating neurite outgrowth and neuron apoptosis [21]. To focus our efforts on elaborating whether PTEN was involved in IS progression as the downstream target of miR-26a-5p, we predicted the potential binding sites between miR-26a-5p and PTEN with the help of the StarBase bioinformatics software and verified the binding relationship in N2a cells by dual-luciferase assay. Additionally, our findings illustrated that PTEN levels in N2a cells were diminished after miR-26a-5p mimic transfection, while the trends were reversed after transfection of miR-26a-5p inhibitor. It is noteworthy that miR-26a-5p protects against I/R-induced myocardial injury by targeting PTEN [39]. In conclusion, miR-26a-5p targeted PTEN. To identify the effects of PTEN on the progression of IS, we knocked down PTEN based on the treatment of sh-circTLK1 and miR-26a-5p inhibitor. Our results noted that PTEN knockdown annulled the effect of miR-26a-5p inhibitor on apoptosis and viability. Consistently, PTEN is raised in N2a cells treated by OGD and overexpression of PTEN attenuates the effects of miR-532-5p on protecting OGD-treated N2a cells [40]. Further in line with our findings, a prior study indicated that miR-26a-5p inhibits apoptosis and protects cardiomyocyte viability against I/R injury by inhibiting PTEN [39]. Collectively, the aforementioned findings and evidence indicated that circTLK1 regulated OGD/R-induced neurological impairment via the miR-26a-5p/PTEN axis.

Existing evidence suggests that PTEN mediates the regulation of IGF-1 R/GLUT1 in cancers [22,23]. PTEN dephosphorylates AKT and inhibits GLUT1 expression on the serosa of cancer cells [23]. On the other hand, IGF-1 R regulates GULT expression by phosphorylating AKT [24]. To verify whether PTEN exerted effects on IS by regulating IGF-1 R/GLUT1, we knocked down PTEN expression. Our results discovered that IGF-1 R and GLUT1 levels in OGD/R-induced N2a cells were decreased after PTEN knockdown. Furthermore, the rescue experiments verified that circTLK1 silencing lowered PTEN, IGF-1 R, and GLUT1 expressions, miR-26a-5p inhibitor upregulated these expressions, while PTEN knockdown annulled the function of miR-26a-5p inhibitor. Finally, we validated the role of circTLK1 in MCAO *in vivo*. Our results denoted that circTLK1 and PTEN expressions were upregulated and miR-26a-5p was suppressed in MCAO mouse brain tissues, while these trends were averted after circTLK1 knockdown, along with reduced IGF-1 R and GLUT1 expressions. The results were consistent with that of *in vivo* experiments. Collectively, our findings indicated that circTLK1 regulated the neurological impairment induced by OGD/R via the miR-26a-5p/PTEN/IGF-1 R/GLUT1 axis.

To conclude, findings obtained in our study supported that knockdown of circTLK1 relieves IS in MCAO mice and OGD/R-induced N2a cells via the miR-26a-5p/PTEN/IGF-1 R/GLUT1 axis. However, this study solely revealed that circTLK1 mediated the pathological process of IS by regulating the miR-26a-5p/PTEN/IGF-1 R/GLUT1 axis, but failed to deeply study how PTEN mediated the IGF-1 R/GLUT1 signal on IS. Further studies are warranted to We shall verify the molecular mechanism of the PTEN-mediated IGF-1 R/GLUT1 signal in regulating IS from the perspective of epigenetics in our future endeavors.

## 5. Conclusion

All in all, circTLK1 was upregulated in IS, which could be used as a ceRNA of miR-26a-5p to regulate the expression of PTEN and promote the progression of IS. Knockdown of circTLK1 alleviated IS by regulating the miR-26a-5p/PTEN/IGF-1 R/GLUT1 axis. Therefore, circTLK1 might be a novel therapeutic target for IS.

## Data Availability

All the data generated or analyzed during this study are included in this published article.
